# The Tobacco Status Project (TSP): Study protocol for a randomized controlled trial of a Facebook smoking cessation intervention for young adults

**DOI:** 10.1186/s12889-015-2217-0

**Published:** 2015-09-15

**Authors:** Danielle E. Ramo, Johannes Thrul, Kevin L. Delucchi, Pamela M. Ling, Sharon M. Hall, Judith J. Prochaska

**Affiliations:** Department of Psychiatry, University of California, 401 Parnassus Avenue, Box TRC 0984, San Francisco, CA 94143 USA; Center for Tobacco Control Research and Education, University of California, 530 Parnassus Avenue, San Francisco, CA 94143 USA; Division of General Internal Medicine, University of California, 1545 Divisadero Street, San Francisco, CA 94143 USA; Department of Medicine, Stanford Prevention Research Center, Stanford University, 1265 Welch Road, Stanford, CA 94305 USA

## Abstract

**Background:**

Tobacco use remains the leading cause of premature morbidity and mortality in the United States. Young adults are less successful at quitting, use cessation treatment less often than smokers of other ages, and can be a challenge to retain in treatment. Social media, integrated into the lives of many young adults, represents a promising strategy to deliver evidence-based smoking cessation treatment to a large, diverse audience. The goal of this trial is to test the efficacy of a stage-based smoking cessation intervention on Facebook for young adults age 18 to 25 on smoking abstinence, reduction in cigarettes smoked, and thoughts about smoking abstinence.

**Methods/Design:**

This is a randomized controlled trial. Young adult smokers throughout the United States are recruited online and randomized to either the 3 month Tobacco Status Project intervention on Facebook or a referral to a smoking cessation website. The intervention consists of assignment to a secret Facebook group tailored to readiness to quit smoking (precontemplation, contemplation, preparation), daily Facebook contacts tailored to readiness to quit smoking, weekly live counseling sessions, and for those in preparation, weekly Cognitive Behavioral Therapy counseling sessions on Facebook. Primary outcome measure is biochemically-verified 7-day point prevalence abstinence from smoking at posttreatment (3 months), 6, and 12 months. Secondary outcome measures are reduction of 50 % or more in cigarettes smoked, 24 h quit attempts, and commitment to abstinence at each time point. A secondary aim is to test, within the TSP condition, the effect of a monetary incentive at increasing engagement in the intervention.

**Discussion:**

This randomized controlled trial is testing a novel Facebook intervention for treating young adults’ tobacco use. If efficacious, the social media intervention could be disseminated widely and expanded to address additional health risks.

**Trial registration:**

ClinicalTrials.gov: NCT02207036, May 13, 2014.

## Background

Cigarette smoking is the single most important preventable cause of morbidity, mortality, and excess health cost in the United States (US), accounting for 480,000 premature deaths each year [1, 2]. Although the prevalence of cigarette smoking has declined among adults in the US since 1983, smoking among young adults aged 18–25 years has remained stable, with past month cigarette use rates as high as 31 % in 2013 [3]. Almost all smokers (98 %) report starting before the age of 26 years [4], and more than 2000 US youth and young adults become daily cigarette smokers each day [4, 5].

Despite comparable smoking prevalence and proportion motivated to quit, relative to other adult age groups, young adults are less likely to use interventions for smoking cessation [6]. Further, studies of tobacco use and other health behaviors have reported great challenges in recruiting young adults [7, 8]. Online smoking cessation strategies (e.g., websites designed to help people quit smoking) reach large numbers of smokers [9] but tend not to have a personalized approach to smoking cessation or to have follow-up treatment contacts [10]. Young adults are less likely to take advantage of these cessation resources, and studies of web-based smoking cessation programs have been associated with large drop-offs in engagement throughout the course of participation [11–14]. Websites directly targeted to young adults have typically focused on college students [15, 16] who are less likely to smoke than non-college bound young adults [17, 18]. There is a need to develop innovative smoking cessation interventions to engage a wide audience of young adult smokers.

Social media represents a promising strategy to deliver evidence-based smoking cessation interventions to young adults. Social media tools are widely popular among young adults (89 % of 18 to 29 year old Americans use the internet) [19] and can be harnessed to widely disseminate information about a broad range of behavioral and emotional changes including smoking cessation [20–23]. Facebook remains the most widely used social media tool by young adults in the United States. With 87 % of US online young adults having a Facebook account and 70 % of those accessing it daily [19], there is promise to use this platform to deliver public health intervention programs to young people. Previous evaluations using Facebook to change health risk behavior have shown feasibility as measured by participant’s engagement and satisfaction [24–32]. However, trials examining social media interventions have shown limited or no effects on health behavior change (e.g., physical activity) [33]. The BIO smoking cessation campaign for young adults in Canada, incorporating a website, Smartphone app and Facebook features, resulted in greater 7- and 30-day reported quit rates than referral to a Smokers’ Helpline at 3 month follow-up [34]. Research is needed to determine whether Facebook alone can be used as an intervention tool, whether abstinence can be biochemically verified, and whether abstinence rates can be maintained over 1 year.

Our group conducted a mixed-methods study to determine how young adult smokers would like to use Facebook to help change smoking behavior. About a third (31 %) of survey respondents reported they would want to quit smoking using Facebook, and interest was greater among those more motivated to quit, who had made a quit attempt in the past year, and had previously used the Internet for assistance with a quit attempt (all *p* < 0.01). In qualitative interviews, social support and convenience were identified as strengths of a Facebook intervention, while privacy was the main concern [35].

Based on this formative work, we developed the *Tobacco Status Project (TSP),* a Facebook intervention for young adult smokers combining Facebook contacts tailored to participants’ readiness to quit smoking with a 12-week cessation program consisting of 90 days of Facebook postings, weekly counseling sessions, and for those ready to quit, 7 state-of-the-art group cognitive-behavioral sessions. A feasibility trial enrolled 79 young adults who formed 7 Facebook groups [36]. Follow-up rates were 84 and 72 % at 6 and 12 months, respectively, and reported 7-day abstinence was 21 % at 6 months (9 % biochemically-verified) and 18 % at 12 months (9 % verified) (Ramo DE, Chavez K, Delucchi KL, Prochaska JJ: Feasibility and quit rates of the “Tobacco Status Project”, submitted). From baseline to 12-months, there was a significant increase in the proportion prepared to quit (13 to 46 %, p < .001), 35 % reduced their cigarette consumption by 50 % or greater, and 67 % reported a 24-h quit attempt. Engagement in the intervention was high, with 92 % participation in the full 3 month intervention and 61 % commented on at least one post, with more commenting among those randomized to receive a personal monetary incentive [37]. Participants reported reading most of the Facebook posts (mean usability rating = 3.3/4) and interactions from counseling sessions (3/4), thinking about what they read (3/4) and would recommend the program to others (3.3/4). Herein, we describe a randomized controlled trial testing the efficacy of TSP against a referral control condition with 480 young adult smokers who use Facebook.

## Aims and hypotheses

### Main Aim: To evaluate the efficacy of the Facebook-based intervention for young adults in a randomized controlled trial

Hypotheses are:

1. Participants receiving the intervention will have higher 7-day point prevalence abstinence at 3-, 6-, and 12-months follow-up compared to those in the control condition.
2. Participants in the intervention condition will demonstrate greater reduction in cigarettes smoked and increased commitment to abstinence at 3-, 6-, and 12-months follow-up than those in the control condition.
3. Participants in the intervention condition will be more likely to make a quit attempt during the 12-month study period compared to those in the control condition.

### Secondary Aim: To evaluate effectiveness of a monetary incentive for engagement in the Tobacco Status Project intervention

Analyses will examine:

1. Engagement by incentive group (daily, weekly, monthly, no incentive);
2. Abstinence rates by incentive group; and
3. Effect of engagement on abstinence

## Methods/Design

### Overview of design

**Fig. 1:**
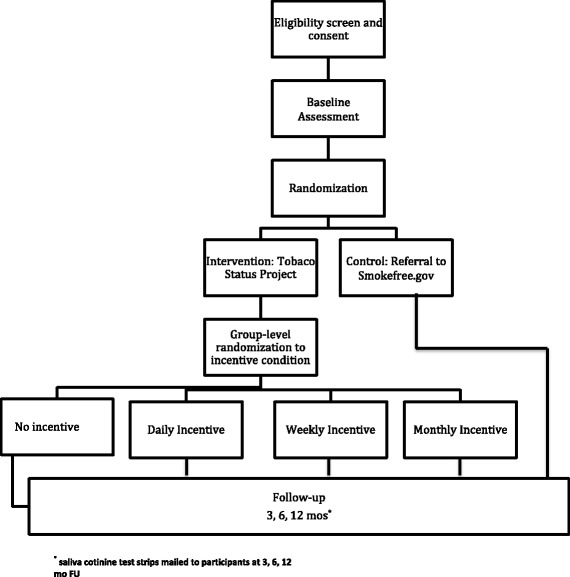
Flow of participants through trial

This study is a randomized controlled trial (RCT) with 480 young adults age 18 to 25 recruited through Facebook (Fig. 1). Participants are randomized to one of two conditions: (1) the Tobacco Status Project motivationally-tailored smoking cessation intervention delivered through Facebook (TSP); or (2) a control condition (referral to the Smokefree.gov website). Assessments are conducted online at baseline, 3-, 6-, and 12-months follow-up. Primary outcome is biochemically-verified 7-day point prevalence abstinence from tobacco, and secondary outcomes are reduction in cigarettes smoked per week, 24 h quit attempts, and commitment to abstinence in each time period. Within the TSP group, a monetary engagement will be tested as a moderator of engagement and abstinence.

### Inclusion criteria

Participants are required to meet the following criteria in order to be eligible for enrollment in the trial:

1. English literate (at an 8^th^ grade reading level);
2. age 18 to 25 years (consistent with the definition of young adulthood used by the National Survey of Drug Use and Health, and age range with the highest smoking prevalence nationally [38]);
3. access to a digital camera that can take and send a picture (e.g., camera phone);
4. go on Facebook “most” (≥4) days per week;
5. ≥100 cigarettes smoked in their lives; and currently smoking at least 1 cigarette per day on 3 or more days of the week. Smoking criteria are based on those used in the National Health Interview Survey and are liberal based on smoking patterns most common in young adults and to include ethnic/racial minority populations, who tend to smoke fewer cigarettes per day than Caucasians [1].

### Exclusion criteria

1. previous participation in the TSP feasibility trial.

### Recruitment

Figure 1 shows the flow of participants through the trial. Participants are being recruited over 16 months primarily from Facebook based on a media campaign that has been successfully employed by the team in previous online survey and interventions studies [36, 39, 40]. Facebook advertisements are targeted based on: 1) age (18 to 25); 2) location; 3) gender; 4) race/ethnicity; and 4) keywords related to tobacco use. Ads include an image and short text consistent with Facebook’s advertising guidelines. Most ads mention the study incentive. Facebook’s Ads Manager program tracks progress of all advertisements in a campaign based on budget, numbers of clicks on an ad, and likelihood of enrollment in a study. Ads include a link to the study’s independent Qualtrics website with a short description of the study and eligibility questions. If respondents are eligible, then they will be taken to the study’s informed consent page.

### Study procedure

Informed consent is obtained from all study participants in accordance with the Helsinki Declaration and approved by the UCSF Committee in Human Research (#11-06294). During the informed consent process, participants’ understanding of the information provided is assessed through a series of multiple choice questions with required responses before being able to proceed with the study. Questions are based on those used in studies with potentially vulnerable populations [41, 42]. Any wrong answers during the consent process are sent to study staff who then contact a potential participant via email or Facebook for further clarification of the consent process. Therefore, no participant will be enrolled that has not answered all consent questions correctly. Participants consenting to participate in the study are asked to verify age by sending proof through email or social media. Verified participants are emailed a baseline assessment. Those completing the baseline assessment are randomized to receive either the Tobacco Status Project (TSP) intervention of referral to the Smokefree.gov website (control).

A stratified random assignment program was developed and participants are assigned from within stratified blocks immediately after completing the baseline assessment. Participants are stratified on stage of change (precontemplation, contemplation, preparation) and smoking pattern (daily vs. non-daily), variables known to be related to outcomes and addressed by the intervention [43]. Participants in the TSP group are assigned to “secret” (private) Facebook groups based on their readiness to quit tobacco. Groups begin when there are approximately 10 participants enrolled, as that has been deemed the optimal number based on analyses of engagement in our previous work [37] (Thrul J, Klein A, Ramo DE: Smoking cessation intervention on Facebook: Which content generates the best engagement?, submitted). Participants in the control condition are given a referral to the Smokefree.gov program and are encouraged to use it as much as they would like.

Assessments occur online using the survey program Qualtrics at baseline, 3, 6, and 12 months follow-up. Participants receive their choice of gift cards in the amount of $20 per assessment, and a $20 bonus for completing all three assessments, for a total possible stipend of $100 for completing all assessments.

### Treatment conditions

#### Tobacco Status Project (TSP)

The TSP is a smoking cessation intervention implemented entirely through “secret” (Facebook’s word for entirely private) Facebook groups. All TSP participants are assigned to a Facebook group based on their TTM stage of change (precontemplation: not ready to quit in the next 6 months; contemplation: intending to quit in the next 6 months; or preparation: ready to quit in the next month and demonstrating at least one past year quit attempt).

**Fig. 2:**
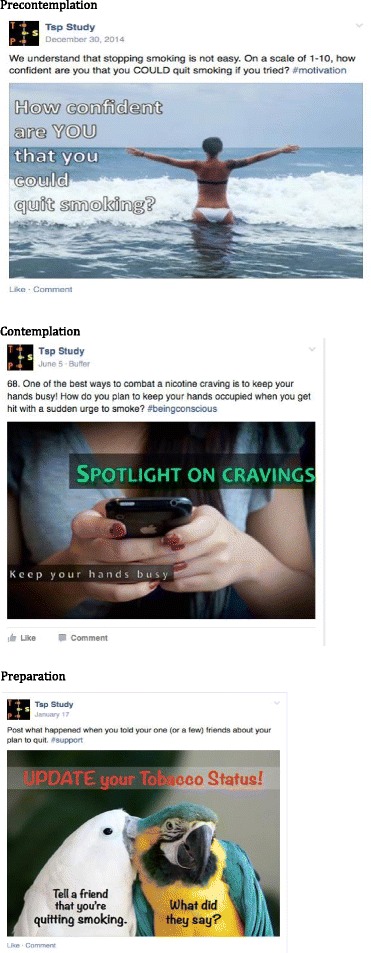
Tobacco Status Project Sample Posts

The group-based intervention has three main features. First, evidence-based strategies have been used to design Facebook posts to be delivered each day for 90 days to intervention groups on Facebook. Posts are based on the US Clinical Practice Guidelines for smoking cessation [2] and the Transtheoretical Model (TTM) of behavior change [44], both recommending treatment be tailored to participants’ readiness to quit, and have been determined to be helpful to and likable by young adults (Ramo DE, Chavez K, Delucchi KL, Prochaska JJ: Feasibility and quit rates of the “Tobacco Status Project”, submitted). Precontemplation group posts are based on Motivational Interviewing (MI), a directive patient-centered counseling intervention recommended by the Clinical Practice Guidelines [2, 45]. Posts elicit clients’ motivation and importance of changing tobacco use, problems associated with use, and using open-ended questions to elicit “change talk” (a client’s mention of desire, ability, reason, or commitment to change) through using the 5-R’s: relevance, risks, rewards, roadblocks, and repetition, shown to increase likelihood of tobacco quit attempts [46, 47]. Contemplation group posts incorporate MI and the TTM processes of self-liberation (e.g., making a commitment to quit), stimulus control (e.g., removing smoking paraphernalia from the home), and counter conditioning (e.g., engaging in alternative behaviors) are emphasized. Preparation group posts incorporate skills from cognitive behavioral therapy, found effective for long-term smoking cessation [48], as well as the TTM processes of self-liberation (e.g., making a commitment to quit), stimulus control (e.g., removing smoking paraphernalia from the home), and counter conditioning (e.g., engaging in alternative behaviors). Posts also encourage setting a quit date and making a detailed quit plan. Facebook posts include a combination of images, videos, text and polls designed to reflect the experience of young adults and all elicit a response from participants. Posts may suggest that participants use their FB or real social networks for support with alcohol or tobacco reduction. However, they are not required to share any information about substance use on social media. Figure 2 provides sample posts for each stage of change.

Second, the intervention incorporates weekly “The Dr. Is In” live sessions with a PhD level smoking cessation counselor (using FB commenting features), during which a counselor provides some limited content for discussion and participants can ask questions and get supplemental support. Content for sessions is tailored to readiness to quit tobacco and is based on Motivational Interviewing (MI) and cognitive behavioral coping skills for smoking cessation.

Third, six manualized 45-min state-of-the-art smoking cessation counseling sessions based on cognitive behavioral therapy (CBT) were adapted for social media delivery for this study. These sessions are designed to be delivered in Facebook events and events are scheduled in each group separately. In contrast to The Dr. Is In sessions that are conversational and question and answer-based, these sessions present more material, and are designed to help participants who are motivated to quit. Thus, only participants in preparation groups are offered these CBT sessions and participants may opt to participate in the sessions at any time during the 90 days of Facebook intervention. In all groups, links to more intensive treatment in a participant’s’ area are available as needed.

Additionally, we are testing an incentive structure in which the TSP groups are randomized to one of four incentive conditions tied to engagement in the intervention: 1) daily; 2) weekly; 3) monthly; or 4) no incentive. Participants in daily, weekly, or monthly groups can earn money based on comments made to daily Facebook posts at the end of each period. At the end of the 90-day intervention, all participants in paid incentive conditions receive a gift card in the amount they have earned up to $90. This is separate from the $100 participants could earn for completing all study assessments.

### Control group

Participants in the control condition, receive a referral to the National Cancer Institute’s Smokefree.gov website. This website, created by the Tobacco Control Research Branch of the National Cancer Institute, provides information and support to quit smoking for people at all stages of change. Smokefree.gov (http://www.smokefree.gov) provides free, accurate, evidence-based information and professional assistance to help support the immediate and long-term needs of people trying to quit smoking. Features of the program include a website tailored to readiness to quit smoking, a texting program, Smartphone application, online live chat, and a Facebook page. The treatment provided to control participants meets U.S. Clinical Practice Guidelines for treating nicotine dependence with adults [49].

### Measures

#### Primary outcome

The primary outcome biochemically-verified 7-day point prevalence abstinence at 3, 6, and 12 months. Participants reporting “no smoking, not even a puff” in the past 7 days will be coded as abstinent from cigarettes. At each follow-up assessment, participants reporting 7-day abstinence will be mailed a saliva cotinine test kit and asked to record two pictures: one giving a saliva sample, and another of the test result. Participants with a salivary cotinine level <11 ng/ml, indicating nonsmoking [50], will be considered confirmed nonsmokers. If participants indicate use of an electronic nicotine delivery-system to aid in smoking cessation, saliva cotinine and reported ENDS use will be recorded and reported separately from biochemically verified abstinence.

### Secondary outcomes

The following secondary outcomes will be assessed at each timepoint:

- **Reduction of cigarette consumption by 50 % or more (y/n)** between baseline each follow-up will be calculated from the number of cigarettes smoked in the past 7 days at each time point.
- **Tobacco quit attempt** (y/n). A *Follow-up Smoking Questionnaire* will assess the presence and number of 24 h quit attempts since the last assessment, used to calculate presence of at least one quit attempt in the assessment time period.
- **Readiness to quit tobacco** will be assessed using the *Stages of Change Questionnaire*, [44] categorizing participants into five stage categories at each timepoint (precontemplation, contemplation, preparation, action, and maintenance), and predictive of quit attempts and cessation [51]. Outcome will be measured as proportion in action or maintenance stage sof change at 3, 6, and 12 month assessments.
- **Abstinence goal** will be assessed with the *Thoughts About Abstinence Form, *[52]*,* categorizing goal as no goal, intermediary goal (e.g., reduced smoking), or total abstinence. Outcome will be measured as proportion endorsing a goal of abstinence at each timepoint.
- **Engagement in TSP** will be measured by total number of comments to Facebook study groups during the three-month intervention period.

### Power calculation

Previous trials of stage-based interventions for smoking cessation [53–57] and trials of Internet interventions for smokers motivated to quit [11, 58] suggest that quit rates for motivated smokers may be as high as 20 % (treatment) and 9 % (control). However, as this will be the first stage-based intervention for young adult smokers delivered over Facebook, and cessation rates in some Internet trials have been as low as 7 % point prevalence abstinence at 3 month follow-up [59], the conservative estimate of 10 % (treatment) and 5 % (control) 7-day point prevalence abstinence at 3, 6, and 12 months follow-up was made. Further, attrition in Internet cessation trials has tended to be as high as 50–75 % at 3 and 6 months follow-up [11, 12, 59]. With 50 % attrition at each time point, and at least one covariate (depending on group differences), a sample size of 480 will provide a minimal power level of .80, and a Type-I error rate of .05 to detect these cessation rates in the most conservative outcome of the study - 7-day point prevalence abstinence.

### Data analysis

#### Primary analyses

Preliminary analyses will describe and summarize all measures and test the correlation between order of study entry and outcome rates of dropout by condition. If differences for any of these variables are noted, they will be statistically controlled as covariates in model testing. Missing data will be minimized through online assessment. When subjects do not complete online assessments, they will be re-contacted through Facebook or email to obtain missing information. For each hypothesis test, two sets of outcome analyses will be conducted - one with all participants who are maintained in the study, and another based only on biochemically verified smoking abstinence rates to allow for direct comparison of findings with the research literature.

The primary outcome of biochemically-verified 7-day abstinence will be compared between participants in the TSP and Control groups at 3- through 12-months follow up using a mixed-effects statistical model [60, 61]. The independent variables will be intervention versus the control condition, assessment point, plus covariates identified in preliminary analysis. The model will be estimated using maximum likelihood estimation.

### Secondary analyses

Mixed effects logistic and multinomial regression models for longitudinal ordinal response data to model secondary outcomes for tobacco use and across time (3, 6, 12 months): 1) reduction of cigarettes by 50 % or more (y/n), 2) tobacco quit attempt (y/n; 2 models), 3) action or maintenance stage of change (y/n); and 4) commitment to abstinence (y/n). Independent variables in all models will be treatment condition, abstinence status, and covariates identified as relevant to smoking characteristics in the literature.

To evaluate the secondary aim (effect of a monetary incentive on engagement in the TSP condition), we will test for differences in engagement and primary smoking outcome by incentive groups and the relationship between engagement and primary smoking outcome at 3 months. First, due to expected skewed distribution of the engagement variable (comments), the non-parametric Kruskal-Wallace test will examine overall differences in total number of comments made to the Facebook study group by incentive condition, with test for a linear trend in commenting by incentive frequency. Second, Pearson’s chi-square test will examine 7-day point prevalence abstinence by incentive condition. Abstinence rates will be examined by incentive group. Two sets of analyses will be conducted: one for biochemically verified abstinence, and one for reported abstinence. Finally, Mann–Whitney U tests will compare comments for those abstinent at 3months to those non-abstinent. Again, two sets of analyses will be conducted (one for biochemically-verified abstinence and one for reported abstinence).

## Discussion

To our knowledge, this is the first clinical trial of a smoking cessation intervention for young adults delivered entirely through Facebook. Intervention through social media is innovative and a particularly good option to reach young adult smokers, given widespread use and integration into the lives of users [19]. Yet intervention using this medium can be complicated with respect to recruitment and study design. For example, formative work with the study population indicated that a substantial minority of young adults, particularly those motivated to quit smoking, would be interested in a smoking cessation intervention delivered through Facebook [35]. Yet, a sample of 79 participants recruited for a feasibility trial was primarily white and male, limiting generalizability of the intervention results [36]. In the present trial, efforts are being made through targeted Facebook advertising to recruit a more diverse participant pool.

In this trial, we chose to compare TSP to a control condition in which participants were referred to the Smokefree.gov website. This remains a state-of-the-art digital treatment-as-usual, given scientific basis of its content, and free online access through multiple digital media. Given the overlap in medium and intervention content, significant results will provide strong evidence for the efficacy of TSP. While the content of TSP is similarly based on the Clinical Practice Guidelines, there are differences in the design and specific material presented, and the all-Facebook delivery is unique to TSP.

A key concern in using Facebook for intervention is participants’ privacy. Social media, by nature, is a public forum for interaction, and there is potential for unintended sharing of information. All TSP intervention components are administered entirely through private groups that are not visible beyond the participants in the groups. Participants are given detailed information about the intervention in the consent process, including notice that all groups are private. However, as in any interaction of social media, data belong to Facebook and their use is dictated by privacy agreements made between each user and Facebook itself, not a research investigator. Participants are made aware during the consent process that their TSP interactions are not completely private and that any concerns should be taken up with the investigators and Facebook itself. All assessments of smoking and other substance use in this study are administered outside of social media (i.e., Qualtrics website housed on a secure UCSF server). The Principal Investigator (first author) has obtained a Federal Certificate of Confidentiality from the NIH to protect all data from subpoena. Investigators have gone to great efforts to ensure that the exchange of information is this trial is used to make positive life changes with the support of an intimate network, and that limitations to privacy and confidentiality are clear to participants.

This trial will help to determine whether and how social media can be harnessed for long-term, biochemically verified smoking cessation in young adults.
